# 2-Amino­pyridinium 5-(5-chloro-2,4-dinitro­phen­yl)-1,3-dimethyl-2,4-dioxo-1,2,3,4-tetra­hydro­pyrimidin-6-olate

**DOI:** 10.1107/S1600536811049518

**Published:** 2011-11-30

**Authors:** Vaduganathan Manickkam, Doraisamyraja Kalaivani

**Affiliations:** a PG & Research Department of Chemistry, Seethalakshmi Ramaswami College, Tiruchirappalli 620 002, Tamil Nadu, India

## Abstract

In the title mol­ecular salt, C_5_H_7_N_2_^+^·C_12_H_8_ClN_4_O_7_^−^, the dihedral angle between the aromatic rings of the anion is 51.88 (6)°. One of the nitro groups is disordered over two orientations in a 0.710 (6):0.290 (6) ratio. In the crystal, the cations and anions are linked by N—H⋯O hydrogen bonds, generating infinite ribbons extending along [100] which incorporate *R*_4_^4^(22) ring motifs. Weak C—H⋯O inter­actions also occur.

## Related literature

For our previous work in this area and background to barbiturate drugs, see: Kalaivani & Buvaneswari (2010 ▶); Kalaivani *et al.* (2008 ▶). For a related structure, see: Swamy *et al.* (2008 ▶).
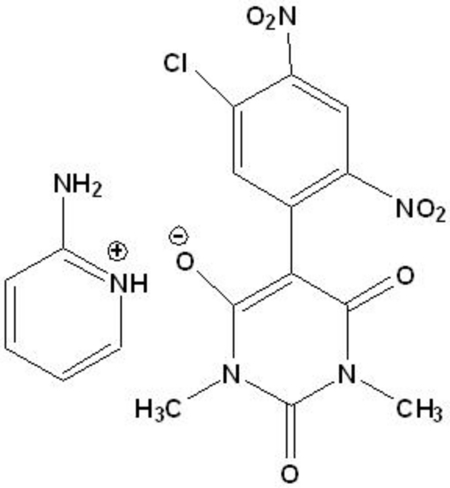


## Experimental

### 

#### Crystal data

C_5_H_7_N_2_^+^·C_12_H_8_ClN_4_O_7_^−^
*M* *_r_* = 450.80
Monoclinic, 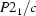

*a* = 8.578 (5) Å
*b* = 11.229 (5) Å
*c* = 19.952 (5) Å
β = 94.952 (5)°
*V* = 1914.7 (15) Å^3^
*Z* = 4
Mo *K*α radiation
μ = 0.26 mm^−1^
*T* = 293 K
0.30 × 0.20 × 0.20 mm

#### Data collection

Bruker Kappa APEXII CCD diffractometer
Absorption correction: multi-scan (*SADABS*; Bruker, 2004 ▶) *T*_min_ = 0.882, *T*_max_ = 0.941
19182 measured reflections
4053 independent reflections
2996 reflections with *I* > 2σ(*I*)
*R*_int_ = 0.032

#### Refinement

*R*[*F* ^2^ > 2σ(*F* ^2^)] = 0.044
*wR*(*F* ^2^) = 0.124
*S* = 1.06
4053 reflections
315 parameters
28 restraints
H atoms treated by a mixture of independent and constrained refinement
Δρ_max_ = 0.38 e Å^−3^
Δρ_min_ = −0.25 e Å^−3^

### 

Data collection: *APEX2* (Bruker, 2004 ▶); cell refinement: *SAINT* (Bruker, 2004 ▶); data reduction: *SAINT*; program(s) used to solve structure: *SIR92* (Altomare *et al.*, 1993 ▶); program(s) used to refine structure: *SHELXL97* (Sheldrick, 2008 ▶); molecular graphics: *ORTEP-3* (Farrugia, 1997 ▶) and *Mercury* (Macrae *et al.*, 2006 ▶); software used to prepare material for publication: *SHELXL97*.

## Supplementary Material

Crystal structure: contains datablock(s) global, I. DOI: 10.1107/S1600536811049518/hb6510sup1.cif

Structure factors: contains datablock(s) I. DOI: 10.1107/S1600536811049518/hb6510Isup2.hkl

Supplementary material file. DOI: 10.1107/S1600536811049518/hb6510Isup3.cml

Additional supplementary materials:  crystallographic information; 3D view; checkCIF report

## Figures and Tables

**Table 1:** Hydrogen-bond geometry (Å, °)

*D*—H⋯*A*	*D*—H	H⋯*A*	*D*⋯*A*	*D*—H⋯*A*
N5—H5*A*⋯O1^i^	0.856 (19)	1.882 (19)	2.730 (3)	170.8 (19)
N6—H6*A*⋯O2^ii^	0.85 (2)	1.976 (19)	2.805 (3)	163 (3)
N6—H6*B*⋯O3	0.85 (2)	2.12 (2)	2.883 (3)	150 (2)
C9—H9⋯O2^iii^	0.93	2.51	3.097 (3)	121
C9—H9⋯O7^iv^	0.93	2.57	3.285 (3)	134
C11—H11*C*⋯O5^v^	0.96	2.59	3.241 (4)	126

Symmetry codes: (i) 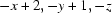; (ii) 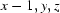; (iii) 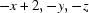; (iv) 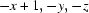; (v) 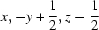.

**Fig. 1.**
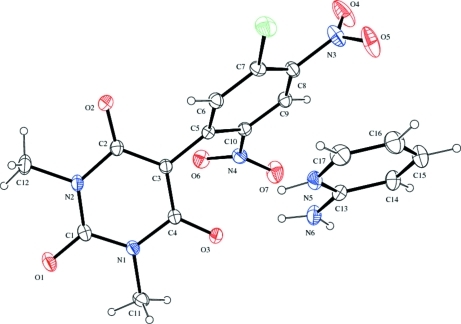
The molecular structure of the title compound showing 30% displacement ellipsoids. Only one orientation of the disordered nitro group is shown.

**Fig. 2.**
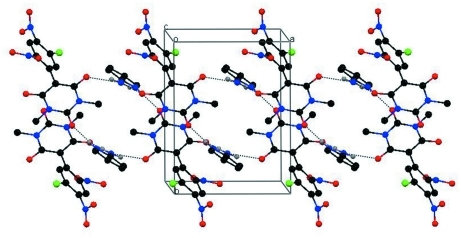
Packing view of (I).

## supplementary crystallographic information

### Comment

As part of our ongoing studies of water soluble barbiturates (Kalaivani *et al.*,2008; Kalaivani & Buvaneswari, 2010), we now report the title barbiturate salt, (I), which has reasonable solubility in polar solvents (water 0.5 g/100 ml; ethanol 1.3 g/100 ml; DMSO 10.1 g/100 ml). As single-crystal X-ray diffraction studies help to understand drug-target interaction (Swamy *et al.*, 2008), the present study may probably help to understand the mechanism of action of barbiturates with biological systems. The cation and anion parts of the title molecule are shown in the Scheme. Fig.1 and Fig.2 are the *ORTEP* and packing views of the title molecular salt respectively. The dihedral angle between the chlorodinitrophenyl ring and 1,3-dimethylbarbiturate ring is observed to be 51.88 (6)°. The nitro group at the *para* position is disordered over two positions with percentage of occupancy 71 and 29.

### Experimental

1,3-Dichloro-4,6-dinitrobenzene (1.18 g, 0.005 mol) was dissolved in 20 ml of absolute alcohol. To this, 0.78 g (0.005 mol) of 1,3-dimethylbarbituric acid was added and the temperature of the mixture was raised to 50°C. To this mixture 1.88 g (0.02 mol) of 2-aminopyridine in 20 ml of absolute alcohol was added. This mixture was shaken well for 2–5 h and kept as such at 25°C for 2 days. On standing, crystals came out from the solution which were filtered and dried. The dry orange colored crystals obtained were powdered well, washed with absolute alcohol and dry ether and then recrystallized from absolute alcohol (yield: 70%; m.pt: 233°C (decomposes at its melting point). Colourless blocks of (I) were obtained by slow evaporation of ethanol at room temperature.

### Refinement

The H atoms of the pyridine nitrogen atoms (H5A, H6A & H6B) were located in difference Fourier maps and refined as riding in their as-found relative positions. The other H atoms were positioned geometrically and were refined using a riding model.

### Crystal data

**Table:**

C_5_H_7_N_2_^+^·C_12_H_8_ClN_4_O_7_^−^	*F*(000) = 928
*M**_r_* = 450.80	*D*_x_ = 1.564 Mg m^−^^3^
Monoclinic, *P*2_1_/*c*	Mo *K*α radiation, λ = 0.71073 Å
Hall symbol: -P 2ybc	Cell parameters from 4951 reflections
*a* = 8.578 (5) Å	θ = 2.4–25.1°
*b* = 11.229 (5) Å	µ = 0.26 mm^−^^1^
*c* = 19.952 (5) Å	*T* = 293 K
β = 94.952 (5)°	Block, colourless
*V* = 1914.7 (15) Å^3^	0.30 × 0.20 × 0.20 mm
*Z* = 4	

### Data collection

**Table:**

Bruker Kappa APEXII CCD diffractometer	4053 independent reflections
Radiation source: fine-focus sealed tube	2996 reflections with *I* > 2σ(*I*)
graphite	*R*_int_ = 0.032
ω and φ scan	θ_max_ = 26.7°, θ_min_ = 2.1°
Absorption correction: multi-scan (*SADABS*; Bruker, 2004)	*h* = −9→10
*T*_min_ = 0.882, *T*_max_ = 0.941	*k* = −14→14
19182 measured reflections	*l* = −18→25

### Refinement

**Table:**

Refinement on *F*^2^	Secondary atom site location: difference Fourier map
Least-squares matrix: full	Hydrogen site location: inferred from neighbouring sites
*R*[*F*^2^ > 2σ(*F*^2^)] = 0.044	H atoms treated by a mixture of independent and constrained refinement
*wR*(*F*^2^) = 0.124	*w* = 1/[σ^2^(*F*_o_^2^) + (0.0563*P*)^2^ + 0.6796*P*] where *P* = (*F*_o_^2^ + 2*F*_c_^2^)/3
*S* = 1.06	(Δ/σ)_max_ = 0.001
4053 reflections	Δρ_max_ = 0.38 e Å^−^^3^
315 parameters	Δρ_min_ = −0.25 e Å^−^^3^
28 restraints	Extinction correction: *SHELXL97* (Sheldrick, 2008), FC^*^=KFC[1+0.001XFC^2^Λ^3^/SIN(2Θ)]^-1/4^
Primary atom site location: structure-invariant direct methods	Extinction coefficient: 0.0051 (11)

### Special details

**Table:**

Geometry. Bond distances, angles *etc*. have been calculated using the rounded fractional coordinates. All su's are estimated from the variances of the (full) variance-covariance matrix. The cell e.s.d.'s are taken into account in the estimation of distances, angles and torsion angles
Refinement. Refinement on *F*^2^ for ALL reflections except those flagged by the user for potential systematic errors. Weighted *R*-factors *wR* and all goodnesses of fit *S* are based on *F*^2^, conventional *R*-factors *R* are based on *F*, with *F* set to zero for negative *F*^2^. The observed criterion of *F*^2^ > σ(*F*^2^) is used only for calculating -*R*-factor-obs *etc*. and is not relevant to the choice of reflections for refinement. *R*-factors based on *F*^2^ are statistically about twice as large as those based on *F*, and *R*-factors based on ALL data will be even larger.

### Fractional atomic coordinates and isotropic or equivalent isotropic displacement parameters (Å^2^)

**Table:**

	*x*	*y*	*z*	*U*_iso_*/*U*_eq_	Occ. (<1)
Cl1	1.03304 (8)	0.08804 (7)	0.22906 (3)	0.0650 (2)	
O1	1.11087 (18)	0.53725 (16)	−0.12650 (8)	0.0532 (6)	
O2	1.24783 (16)	0.23068 (14)	0.01566 (8)	0.0460 (5)	
O3	0.72232 (15)	0.33672 (15)	−0.03589 (7)	0.0459 (5)	
O4	0.7709 (6)	−0.1850 (3)	0.15232 (19)	0.1030 (18)	0.710 (6)
O5	0.7145 (5)	−0.0500 (3)	0.22061 (16)	0.0947 (14)	0.710 (6)
O6	0.8277 (2)	0.09835 (17)	−0.09732 (8)	0.0562 (6)	
O7	0.6060 (2)	0.0610 (2)	−0.05967 (10)	0.0784 (8)	
N1	0.91735 (19)	0.43435 (15)	−0.08264 (8)	0.0365 (5)	
N2	1.17873 (18)	0.38319 (16)	−0.05623 (8)	0.0388 (5)	
N3	0.7806 (3)	−0.0840 (2)	0.17169 (11)	0.0648 (9)	
N4	0.7452 (2)	0.08402 (17)	−0.05157 (9)	0.0448 (6)	
C1	1.0719 (2)	0.4558 (2)	−0.09035 (10)	0.0381 (6)	
C2	1.1398 (2)	0.29008 (19)	−0.01345 (10)	0.0365 (6)	
C3	0.9800 (2)	0.27172 (18)	−0.00748 (10)	0.0350 (6)	
C4	0.8653 (2)	0.34440 (18)	−0.04092 (9)	0.0351 (6)	
C5	0.9311 (2)	0.17671 (18)	0.03654 (10)	0.0345 (6)	
C6	0.9943 (2)	0.1711 (2)	0.10340 (10)	0.0398 (6)	
C7	0.9474 (2)	0.0883 (2)	0.14804 (10)	0.0408 (7)	
C8	0.8320 (2)	0.00736 (19)	0.12644 (11)	0.0423 (7)	
C9	0.7662 (2)	0.00879 (19)	0.06162 (11)	0.0417 (7)	
C10	0.8161 (2)	0.09193 (18)	0.01787 (10)	0.0355 (6)	
C11	0.8007 (3)	0.5095 (2)	−0.11964 (11)	0.0456 (7)	
C12	1.3446 (2)	0.4019 (2)	−0.06582 (13)	0.0545 (8)	
O4'	0.6703 (16)	−0.1204 (15)	0.1656 (8)	0.152 (5)	0.290 (6)
O5'	0.8902 (10)	−0.1336 (8)	0.2104 (5)	0.102 (3)	0.290 (6)
N5	0.6809 (2)	0.31348 (18)	0.17967 (10)	0.0481 (6)	
N6	0.5498 (2)	0.2728 (2)	0.07658 (10)	0.0532 (7)	
C13	0.5615 (2)	0.2616 (2)	0.14244 (11)	0.0400 (7)	
C14	0.4528 (3)	0.1982 (2)	0.17685 (12)	0.0495 (8)	
C15	0.4704 (3)	0.1919 (3)	0.24481 (13)	0.0594 (9)	
C16	0.5962 (3)	0.2470 (3)	0.28138 (13)	0.0619 (9)	
C17	0.6990 (3)	0.3063 (2)	0.24771 (13)	0.0575 (9)	
H6	1.07120	0.22570	0.11820	0.0480*	
H11B	0.71160	0.46200	−0.13510	0.0680*	
H11C	0.84510	0.54440	−0.15760	0.0680*	
H12A	1.35390	0.43670	−0.10920	0.0820*	
H12B	1.39840	0.32690	−0.06300	0.0820*	
H12C	1.39000	0.45440	−0.03150	0.0820*	
H9	0.68870	−0.04570	0.04740	0.0500*	
H11A	0.76860	0.57140	−0.09060	0.0680*	
H5A	0.744 (2)	0.3561 (18)	0.1589 (10)	0.049 (7)*	
H6A	0.467 (2)	0.249 (3)	0.0536 (13)	0.073 (9)*	
H6B	0.623 (2)	0.302 (2)	0.0556 (12)	0.069 (8)*	
H14	0.36870	0.16050	0.15310	0.0590*	
H15	0.39740	0.15020	0.26750	0.0710*	
H16	0.60800	0.24250	0.32810	0.0740*	
H17	0.78410	0.34320	0.27120	0.0690*	

### Atomic displacement parameters (Å^2^)

**Table:**

	*U*^11^	*U*^22^	*U*^33^	*U*^12^	*U*^13^	*U*^23^
Cl1	0.0728 (4)	0.0825 (5)	0.0377 (3)	−0.0051 (3)	−0.0068 (3)	0.0101 (3)
O1	0.0544 (9)	0.0594 (11)	0.0458 (9)	−0.0187 (8)	0.0043 (7)	0.0157 (8)
O2	0.0325 (7)	0.0504 (9)	0.0544 (9)	−0.0007 (6)	0.0004 (6)	0.0074 (8)
O3	0.0312 (7)	0.0556 (10)	0.0516 (9)	0.0007 (7)	0.0071 (6)	0.0137 (8)
O4	0.186 (4)	0.0408 (19)	0.091 (3)	−0.016 (2)	0.063 (3)	0.0045 (17)
O5	0.149 (3)	0.082 (2)	0.0616 (19)	−0.041 (2)	0.058 (2)	−0.0098 (17)
O6	0.0605 (10)	0.0705 (12)	0.0379 (9)	−0.0008 (8)	0.0061 (7)	−0.0040 (8)
O7	0.0489 (10)	0.1158 (18)	0.0668 (12)	−0.0312 (11)	−0.0159 (8)	0.0137 (12)
N1	0.0365 (8)	0.0383 (10)	0.0345 (9)	−0.0047 (7)	0.0023 (7)	0.0049 (8)
N2	0.0319 (8)	0.0457 (11)	0.0397 (9)	−0.0083 (7)	0.0079 (7)	0.0043 (8)
N3	0.0844 (17)	0.0585 (16)	0.0519 (14)	−0.0244 (13)	0.0078 (12)	0.0123 (12)
N4	0.0436 (10)	0.0453 (11)	0.0443 (10)	−0.0049 (8)	−0.0024 (8)	0.0028 (9)
C1	0.0434 (11)	0.0418 (12)	0.0292 (10)	−0.0116 (9)	0.0035 (8)	−0.0015 (9)
C2	0.0334 (10)	0.0394 (12)	0.0368 (11)	−0.0061 (8)	0.0030 (8)	−0.0013 (9)
C3	0.0322 (10)	0.0380 (11)	0.0347 (10)	−0.0040 (8)	0.0027 (8)	0.0035 (9)
C4	0.0349 (10)	0.0370 (11)	0.0338 (10)	−0.0045 (8)	0.0049 (8)	0.0015 (9)
C5	0.0285 (9)	0.0361 (11)	0.0390 (11)	0.0009 (8)	0.0040 (8)	0.0023 (9)
C6	0.0370 (10)	0.0408 (12)	0.0408 (11)	−0.0062 (9)	−0.0003 (8)	0.0027 (10)
C7	0.0460 (11)	0.0416 (12)	0.0345 (11)	0.0016 (9)	0.0015 (9)	0.0038 (10)
C8	0.0479 (12)	0.0378 (12)	0.0426 (12)	−0.0017 (9)	0.0113 (9)	0.0075 (10)
C9	0.0379 (10)	0.0377 (12)	0.0495 (12)	−0.0060 (9)	0.0047 (9)	0.0010 (10)
C10	0.0330 (9)	0.0382 (11)	0.0349 (10)	−0.0002 (8)	0.0011 (8)	0.0017 (9)
C11	0.0496 (12)	0.0420 (13)	0.0441 (12)	−0.0024 (10)	−0.0028 (9)	0.0092 (10)
C12	0.0361 (11)	0.0690 (17)	0.0598 (15)	−0.0140 (11)	0.0121 (10)	0.0068 (13)
O4'	0.117 (7)	0.166 (9)	0.170 (9)	−0.057 (7)	−0.003 (6)	0.078 (8)
O5'	0.096 (5)	0.092 (6)	0.116 (6)	0.000 (4)	−0.007 (4)	0.078 (5)
N5	0.0361 (9)	0.0528 (12)	0.0555 (12)	−0.0102 (8)	0.0046 (8)	0.0082 (10)
N6	0.0390 (11)	0.0754 (15)	0.0458 (12)	−0.0071 (10)	0.0080 (9)	0.0075 (11)
C13	0.0328 (10)	0.0425 (12)	0.0451 (12)	0.0019 (9)	0.0066 (8)	0.0022 (10)
C14	0.0414 (11)	0.0566 (15)	0.0512 (14)	−0.0118 (10)	0.0081 (10)	−0.0022 (12)
C15	0.0610 (15)	0.0685 (18)	0.0508 (14)	−0.0150 (13)	0.0166 (12)	0.0058 (13)
C16	0.0702 (16)	0.0725 (18)	0.0429 (13)	−0.0057 (14)	0.0048 (12)	0.0003 (13)
C17	0.0535 (14)	0.0628 (17)	0.0542 (15)	−0.0091 (12)	−0.0064 (11)	−0.0014 (13)

### Geometric parameters (Å, °)

**Table:**

Cl1—C7	1.717 (2)	C3—C4	1.402 (3)
O1—C1	1.229 (3)	C3—C5	1.466 (3)
O2—C2	1.244 (3)	C5—C6	1.397 (3)
O3—C4	1.242 (2)	C5—C10	1.398 (3)
O4—N3	1.199 (4)	C6—C7	1.372 (3)
O4'—N3	1.028 (15)	C7—C8	1.385 (3)
O5—N3	1.231 (4)	C8—C9	1.366 (3)
O5'—N3	1.290 (10)	C9—C10	1.372 (3)
O6—N4	1.213 (3)	C6—H6	0.9300
O7—N4	1.219 (3)	C9—H9	0.9300
N1—C4	1.406 (3)	C11—H11C	0.9600
N1—C1	1.369 (3)	C11—H11A	0.9600
N1—C11	1.460 (3)	C11—H11B	0.9600
N2—C12	1.467 (2)	C12—H12A	0.9600
N2—C2	1.408 (3)	C12—H12B	0.9600
N2—C1	1.364 (3)	C12—H12C	0.9600
N3—C8	1.460 (3)	C13—C14	1.399 (3)
N4—C10	1.467 (3)	C14—C15	1.353 (4)
N5—C13	1.345 (3)	C15—C16	1.394 (4)
N5—C17	1.355 (3)	C16—C17	1.332 (4)
N6—C13	1.315 (3)	C14—H14	0.9300
N5—H5A	0.856 (19)	C15—H15	0.9300
N6—H6B	0.85 (2)	C16—H16	0.9300
N6—H6A	0.85 (2)	C17—H17	0.9300
C2—C3	1.401 (3)		
			
C1—N1—C4	123.57 (17)	C6—C7—C8	118.99 (19)
C1—N1—C11	118.01 (17)	N3—C8—C7	121.0 (2)
C4—N1—C11	118.42 (16)	N3—C8—C9	118.15 (19)
C1—N2—C2	124.21 (16)	C7—C8—C9	120.85 (19)
C1—N2—C12	117.82 (17)	C8—C9—C10	118.98 (18)
C2—N2—C12	117.96 (16)	C5—C10—C9	123.07 (19)
O4—N3—O5	121.7 (3)	N4—C10—C5	121.22 (18)
O4—N3—C8	118.8 (3)	N4—C10—C9	115.67 (17)
O5—N3—C8	117.2 (2)	C5—C6—H6	119.00
O4'—N3—C8	122.0 (9)	C7—C6—H6	119.00
O5'—N3—C8	115.5 (4)	C10—C9—H9	121.00
O4'—N3—O5'	121.0 (10)	C8—C9—H9	120.00
O6—N4—O7	123.84 (19)	H11A—C11—H11C	110.00
O6—N4—C10	118.78 (17)	N1—C11—H11A	109.00
O7—N4—C10	117.37 (17)	N1—C11—H11B	109.00
C13—N5—C17	122.9 (2)	H11A—C11—H11B	110.00
C17—N5—H5A	119.7 (13)	H11B—C11—H11C	109.00
C13—N5—H5A	117.3 (13)	N1—C11—H11C	109.00
C13—N6—H6B	122.2 (15)	H12B—C12—H12C	109.00
C13—N6—H6A	119.6 (17)	H12A—C12—H12C	109.00
H6A—N6—H6B	118 (2)	N2—C12—H12A	109.00
O1—C1—N1	120.80 (18)	N2—C12—H12B	109.00
N1—C1—N2	117.01 (18)	N2—C12—H12C	109.00
O1—C1—N2	122.19 (17)	H12A—C12—H12B	109.00
O2—C2—N2	118.36 (16)	N5—C13—C14	117.2 (2)
O2—C2—C3	125.17 (19)	N6—C13—C14	122.84 (19)
N2—C2—C3	116.47 (17)	N5—C13—N6	119.95 (19)
C4—C3—C5	118.91 (16)	C13—C14—C15	119.8 (2)
C2—C3—C4	121.69 (18)	C14—C15—C16	121.0 (3)
C2—C3—C5	119.38 (17)	C15—C16—C17	118.3 (2)
O3—C4—C3	125.33 (18)	N5—C17—C16	120.8 (2)
O3—C4—N1	117.64 (17)	C13—C14—H14	120.00
N1—C4—C3	117.02 (16)	C15—C14—H14	120.00
C3—C5—C10	124.59 (18)	C14—C15—H15	120.00
C6—C5—C10	115.49 (18)	C16—C15—H15	119.00
C3—C5—C6	119.82 (17)	C15—C16—H16	121.00
C5—C6—C7	122.62 (18)	C17—C16—H16	121.00
Cl1—C7—C8	121.88 (17)	N5—C17—H17	120.00
Cl1—C7—C6	119.14 (15)	C16—C17—H17	120.00
			
C4—N1—C1—O1	−178.55 (19)	O2—C2—C3—C5	0.7 (3)
C11—N1—C1—O1	1.2 (3)	C2—C3—C4—O3	−176.86 (19)
C4—N1—C1—N2	1.4 (3)	C4—C3—C5—C10	50.9 (3)
C11—N1—C1—N2	−178.89 (18)	C2—C3—C5—C10	−131.1 (2)
C1—N1—C4—C3	−1.9 (3)	C4—C3—C5—C6	−125.3 (2)
C11—N1—C4—C3	178.34 (18)	C5—C3—C4—O3	1.1 (3)
C1—N1—C4—O3	177.10 (18)	C2—C3—C5—C6	52.7 (3)
C11—N1—C4—O3	−2.6 (3)	C2—C3—C4—N1	2.1 (3)
C1—N2—C2—O2	−179.13 (19)	C5—C3—C4—N1	−179.97 (18)
C2—N2—C1—N1	−1.0 (3)	C6—C5—C10—N4	−177.07 (17)
C12—N2—C1—N1	177.76 (18)	C3—C5—C10—N4	6.6 (3)
C2—N2—C1—O1	178.9 (2)	C3—C5—C10—C9	−175.78 (18)
C12—N2—C1—O1	−2.3 (3)	C6—C5—C10—C9	0.5 (3)
C12—N2—C2—C3	−177.59 (19)	C10—C5—C6—C7	0.0 (3)
C12—N2—C2—O2	2.2 (3)	C3—C5—C6—C7	176.49 (18)
C1—N2—C2—C3	1.1 (3)	C5—C6—C7—Cl1	179.60 (16)
O5—N3—C8—C9	−116.6 (3)	C5—C6—C7—C8	−0.5 (3)
O4—N3—C8—C9	46.4 (4)	C6—C7—C8—C9	0.5 (3)
O5—N3—C8—C7	65.2 (3)	Cl1—C7—C8—C9	−179.60 (16)
O4—N3—C8—C7	−131.8 (3)	C6—C7—C8—N3	178.68 (19)
O6—N4—C10—C5	38.9 (3)	Cl1—C7—C8—N3	−1.4 (3)
O7—N4—C10—C5	−142.5 (2)	N3—C8—C9—C10	−178.23 (19)
O7—N4—C10—C9	39.8 (3)	C7—C8—C9—C10	0.0 (3)
O6—N4—C10—C9	−138.9 (2)	C8—C9—C10—C5	−0.5 (3)
C13—N5—C17—C16	−0.6 (4)	C8—C9—C10—N4	177.19 (17)
C17—N5—C13—C14	0.1 (3)	N5—C13—C14—C15	0.4 (3)
C17—N5—C13—N6	179.4 (2)	N6—C13—C14—C15	−178.9 (2)
N2—C2—C3—C5	−179.65 (17)	C13—C14—C15—C16	−0.5 (4)
O2—C2—C3—C4	178.6 (2)	C14—C15—C16—C17	0.0 (5)
N2—C2—C3—C4	−1.7 (3)	C15—C16—C17—N5	0.5 (4)

### Hydrogen-bond geometry (Å, °)

**Table:**

*D*—H···*A*	*D*—H	H···*A*	*D*···*A*	*D*—H···*A*
N5—H5A···O1^i^	0.856 (19)	1.882 (19)	2.730 (3)	170.8 (19)
N6—H6A···O2^ii^	0.85 (2)	1.976 (19)	2.805 (3)	163 (3)
N6—H6B···O3	0.85 (2)	2.12 (2)	2.883 (3)	150 (2)
C9—H9···O2^iii^	0.93	2.51	3.097 (3)	121
C9—H9···O7^iv^	0.93	2.57	3.285 (3)	134
C11—H11C···O5^v^	0.96	2.59	3.241 (4)	126

Symmetry codes: (i) −*x*+2, −*y*+1, −*z*; (ii) *x*−1, *y*, *z*; (iii) −*x*+2, −*y*, −*z*; (iv) −*x*+1, −*y*, −*z*; (v) *x*, −*y*+1/2, *z*−1/2.
